# Supplementary material for: Dynamic ice–ocean pathways along the Transpolar Drift amplify the dispersal of Siberian matter
Source: Nat Commun. 2025 Apr 14;16:3172. doi: 10.1038/s41467-025-57881-9 (PMC11997031; doi:10.1038/s41467-025-57881-9)
Supplement: Supplementary file 1 — Supplementary Information [file 41467_2025_57881_MOESM1_ESM.pdf]

Supplementary Information to

Dynamic Ice–Ocean Pathways along the Transpolar Drift Amplify the Dispersal of Siberian Matter

Georgi Laukert[1-5*], Dorothea Bauch[5,6], Benjamin Rabe[7], Thomas Krumpen[7], Ellen Damm[7], Markus Kienast[4], Ed Hathorne[5], Myriel Vredenborg[7], Sandra Tippenhauer[7], Nils Andersen[6], Hanno Meyer[8], Moein Mellat[8], Alessandra D'Angelo[9], Patric Simões Pereira[10,11], Daiki Nomura[12], Tristan J. Horner[2,3], Katharine Hendry[1,13], Stephanie S. Kienast[4]

[1] School of Earth Sciences, University of Bristol, Bristol, United Kingdom
[2] Department of Marine Chemistry & Geochemistry, Woods Hole Oceanographic Institution, Woods Hole, USA
[3] NIRVANA Labs, Woods Hole Oceanographic Institution, Woods Hole, USA
[4] Department of Oceanography, Dalhousie University, Halifax, Canada
[5] GEOMAR Helmholtz Centre for Ocean Research, Kiel, Germany
[6] Christian-Albrechts University of Kiel, Kiel, Germany
[7] Alfred-Wegener-Institut, Helmholtz-Zentrum für Polar- und Meeresforschung, Bremerhaven, Germany
[8] Alfred-Wegener-Institut, Helmholtz-Zentrum für Polar- und Meeresforschung, Potsdam, Germany
[9] University of Rhode Island, Graduate School of Oceanography, Narragansett, USA
[10] Department of Marine Sciences, University of Gothenburg, Gothenburg, Sweden
[11] Present address: Institute of Geology, University of Innsbruck, Innsbruck, Austria
[12] Hokkaido University, Hakodate, Japan
[13] British Antarctic Survey, High Cross, Madingley Road, Cambridge, UK
* Corresponding author: Georgi Laukert (georgi.laukert@bristol.ac.uk)

Supplementary Text 1:

Influence of non-conservative processes on dissolved neodymium concentrations and isotopes

Seawater–particle interactions can significantly influence dissolved neodymium (Nd) concentrations ([Nd]) and isotopic compositions (ε_{Nd}) beyond what can be expected from conservative behavior associated with external inputs and water mass advection and mixing. Accounting for these interactions is crucial for our interpretations, as both parameters are integrated in our water mass analysis. While salt-induced coagulation of nanoparticles and colloids in estuaries removes Nd without affecting ε_{Nd}, the release of Nd from particles or the exchange between particulate and dissolved phases—the so-called "boundary exchange"—can significantly alter ε_{Nd} values, sometimes without notable changes in [Nd]. Although boundary exchange is challenging to detect, it has been suggested to occur in various marine settings through multiple mechanisms[1]. In our study of river water pathways, understanding ε_{Nd} alteration due to weathering of suspended or deposited riverine particles is particularly pertinent. The most compelling evidence for this process comes from the Amazon estuary, where shifts to less radiogenic ε_{Nd} compositions at higher salinities have been attributed to continued weathering of suspended particles during estuarine mixing[2]. However, a recent study by Xu et al.[3] challenges this view, showing that ε_{Nd} variations in the Amazon estuary are instead caused by mixing with water from the neighboring Pará River. This reevaluation highlights the ongoing debate on the occurrence and significance of boundary exchange in estuarine systems.

Although clear evidence is lacking, concerns persist about the potential impact of continued weathering of riverine particles on dissolved ε_{Nd}, especially in the Arctic Ocean[4-6]. Nearly half of the Arctic Ocean comprises shallow shelves that receive substantial input from rivers, favoring seawater–particle interactions that could significantly alter ε_{Nd} signatures. However, studies examining the distribution of dissolved rare earth elements (REEs), including Nd, in the river-influenced regions of the Laptev[6], White[7] and Barents[8] seas show no significant REE release from river-borne particles or shelf sediments. Furthermore, water samples collected directly above the sediment surface (multicorer water) in the Laptev Sea reveal no signs of ε_{Nd} alteration due to REE release from or exchange with sediments[6]. The only non-conservative process observed in these regions is the above-mentioned estuarine removal of dissolved REEs, which does not affect dissolved ε_{Nd}. This process occurs at a slower rate compared to other estuaries[6], indicating fundamentally different riverine and estuarine dynamics that may also explain the limited seawater–particle exchange observed. The Siberian rivers are characterized by very low amounts of suspended particulate matter[9,10], which, in combination with a high ratio of organic to inorganic nanoparticles and colloids[7,11], greatly reduces the potential for significant exchange between seawater and particles or the release of particulate REEs[12,13]. Although minor interactions with suspended or deposited sediments in Siberian estuaries cannot be completely ruled out, they are not expected to substantially alter the dissolved ε_{Nd} signatures of river water. This is supported by the strong resemblance between the riverbed surface ε_{Nd} values of the Lena (-14.8) and the Yenisei/Ob (-6.4 to -8.2) rivers[14] and their dissolved ε_{Nd} signatures[6,15,16].

Further downstream in the Transpolar Drift (TPD), significant non-conservative changes in dissolved ε_{Nd} are also unlikely. Studies indicate that dissolved REE concentrations in Siberian-sourced waters traversing the Arctic Ocean and exiting via the Fram Strait remain largely stable throughout their journey[5,6,17,18]. Although the residence time of Nd in the surface ocean is shorter than in the deep open ocean, it still exceeds the estimated transport time of ice and water along the TPD, which is less than 10 years[18-21]. This stability is crucial for using ε_{Nd} as a water mass tracer. The perennial ice cover in the central Arctic plays a significant role in maintaining concentrations by limiting biological productivity and REE cycling[22]. The reduced particle flux and low biological activity minimize seawater-particle exchange, which even leads to uniform vertical REE profiles—a distinctive characteristic of the Arctic Ocean[23]. As a result, all dissolved ε_{Nd} signatures reported for the Eurasian Basin to date are attributable to external inputs of dissolved Nd and conservative mixing (see Supplementary Fig. S4). The strong correlation between dissolved [Nd] and the river water fraction observed in our study (Fig. 4a, main text) reinforces this observation, reflecting the gradual, density-driven merging of Siberian river waters—characterized by distinct ε_{Nd} compositions but very similar [Nd]—with Atlantic water (see also Supplementary Text 2.4). In contrast, non-conservative REE inputs have been suggested to influence the dissolved ε_{Nd} composition of Pacific water emerging from the Chukchi Sea[24,25]. While these changes can be addressed through endmember selection, they are not relevant for our study, as our study region lies well outside the influence of Pacific water (see Supplementary Text 2.4).

In the Fram Strait, the exit region of the TPD, non-conservative processes associated with Greenland inputs have been proposed to locally alter dissolved ε_{Nd} signatures and [Nd][5]. Bottom waters of the NE Greenland Shelf exhibit ε_{Nd} values resembling AW, but with significantly higher [Nd] (up to 24.0 pmol kg^{-1}), which cannot be explained by AW advection or mixing between AW and surrounding water masses. Laukert et al.[5] attributed the less radiogenic ε_{Nd} and elevated [Nd] to remineralization of biogenic particles from unradiogenic shelf surface waters. Later, these changes have been associated with the dissolution of inorganic particles originating directly from Greenland[26]. Although the ε_{Nd} composition of particles and surface sediments on the NE Greenland Shelf is unknown, it is likely highly unradiogenic, consistent with

the unradiogenic nature of rocks from NE Greenland[5,27]. One sample from our Fram Strait station (PS122/4_49-14, 100 m) appears to have been influenced by REE inputs from Greenland, as it deviates from the compositional range of other samples towards less radiogenic ε_{Nd} values (Fig. 4b, main text). It remains unclear whether this deviation is due to the non-conservative processes described above or reflects conservative mixing with Greenland meltwater (Supplementary Text 2.2). Regardless of the cause, we have excluded this sample from our water mass analysis.

We conclude that the estuarine removal of dissolved Nd is the only non-conservative process impacting our water mass analysis. To account for this process, we adjusted the [Nd] values of the two river endmembers to reflect removal rates of 70-80% (Supplementary Text 2.2). To minimize bias, we used the Nd concentrations solely in the isotopic balance to calculate the concentration-weighted average of the isotopic composition (see Methods section of the main text). This approach contrasts with that of Paffrath et al.[17], who incorporated an Nd concentration balance, which introduced sensitivity issues and compromised their analysis. Through exclusion of the measured [Nd] of the samples from our water mass analysis, we can assess the robustness of our method by comparing these values with those predicted from conservative endmember mixing. For most samples, the measured and predicted [Nd] values align within error (Supplementary Fig. S5). However, at higher [Nd], a weak trend towards lower measured values is observed, indicating either slightly higher removal rates than expected (>80 %) or a minor contribution from other Siberian rivers with lower [Nd] endmember values, such as the Kolyma River, which has a removal-uncorrected [Nd] value of 129 pmol kg^{-1} (Supplementary Text 2.2). In either case, our conclusions remain largely unaffected.

Supplementary Text 2:

Selection and characterization of endmembers for water mass analysis

The salinity, $\delta^{18}O$, [Nd], and ε_{Nd} compositions of the endmembers used in this study are summarized in Supplementary Table S1. Below, we outline the rationale for selecting these endmembers—Atlantic water (section 2.1), the fresh waters of the Lena, Yenisei, and Ob rivers (section 2.2), and sea ice meltwater or brine (section 2.3)—while excluding Pacific water (section 2.4). This selection largely builds upon the foundational work of Laukert et al.[5,6,8] and Paffrath et al.[17].

2.1 Atlantic-sourced water

Atlantic Water (AW) is characterized as warm and saline water from the North Atlantic entering the Arctic Ocean through the eastern Fram Strait and the western Barents Sea, with potential density ranging between 27.70 and 27.97 and potential temperatures exceeding 2 °C[28]. AW is the dominant water mass throughout the water column in the Eurasian Basin and is thus a key component in our water mass analysis. We derived the salinity, [Nd], and ε_{Nd} values primarily from the averages reported by Laukert et al.[5,8]. Additionally, for [Nd], we incorporated the lowest observed value (11 pmol kg^{-1}) from Barents Sea Arctic Atlantic Water to account for potential Nd removal processes that occur as AW passes through the relatively productive Barents Sea[8]. The $\delta^{18}O$ range for AW was adopted from Rosén et al.[29] and accounts for slight deviations from the widely used value of 0.3 ‰[30,31].

Laukert et al.[5] documented a gradual shift in ε_{Nd} values as AW traverses the Arctic Ocean and Nordic Seas, raising concerns about its potential impact on the ε_{Nd} characterization of the AW endmember. The shift from unradiogenic signatures (−13) at the Greenland–Scotland Ridge to more radiogenic values (−9) in the Canada Basin corresponds to a decrease in both temperature and salinity within the AW layer. These changes reflect the transformation of AW into colder and less saline Arctic Atlantic Water (AAW), driven by heat loss from ice melt, atmospheric exchange, and mixing with colder and fresher waters from the Barents and Chukchi seas, as well as river runoff[32]. In the Eurasian Basin, our study area, Nd isotopes indicate that these changes are primarily due to the addition of dense waters from the Kara Sea, which contain small amounts of fresh water from the Yenisei and Ob rivers[5,8]. Since we have defined the Yenisei and Ob rivers as an endmember in our water mass analysis (Supplementary Text 2.2), this ε_{Nd} shift is accounted for. Consequently, we can exclude the more radiogenic signatures of AAW observed along the Arctic Circumpolar Boundary Current from our characterization of the (A)AW endmember.

2.2 River water and glacial meltwater

The Lena, Yenisei, and Ob rivers collectively account for approximately 40% of the total riverine discharge to the Arctic Ocean[33], exerting a significant influence particularly on the Eurasian Basin. Consequently, we include these rivers as endmembers in our water mass analysis. We used compositional ranges for $\delta^{18}O$ from Rosén et al.[29] and Cooper et al.[34], as well as for [Nd] and ε_{Nd} from Laukert et al.[6], Zimmermann et al.[15], and Persson et al.[16]. The [Nd] values were adjusted to reflect a 70–80% removal rate in estuaries, as surface waters exported from the Laptev Sea with salinities greater than 25 exhibit Nd removal within this range[6]. This adjustment offers a more accurate representation of riverine Nd inputs as they transition into the open Arctic Ocean.

While smaller Siberian rivers contribute to the TPD, they are indistinguishable from the larger Siberian rivers in our analysis due to similar ε_{Nd} signatures resulting from the drainage of the same lithologies. For

instance, the Khatanga River drains the same young basalts of the Putorana Plateau as the Yenisei and Ob rivers and likely has a very similar ε_{Nd} composition[6]. Nonetheless, these smaller rivers are included in our analysis in terms of river water volume due to their zero salinities, as well as their comparable $\delta^{18}O$[34,35] and [Nd] (e.g., 129 pmol kg^{-1} for the Kolyma River[4]) endmember values to those of the Lena, Yenisei, and Ob rivers. Although their contributions are relatively minor, they enhance the variability in river water transport along the TPD, reinforcing our overall findings. In contrast, the influence of most Canadian rivers on the TPD appears negligible, as they primarily discharge into the Canadian Arctic Archipelago. The plume from the Mackenzie River, North America's largest river flowing directly into the Arctic Ocean, predominantly spreads along the Canadian coastline, extending only 200–400 km into the Beaufort Sea[36,37], indicating a minimal contribution to the TPD. This is supported by a recent analysis of low salinity lenses in the Arctic Ocean, which suggests that the Siberian Shelf is a major source region of fresh water encountered in the Eurasian Basin[38].

Significant glacial meltwater input in our study area is primarily expected from East Greenland, although its influence is likely restricted to the region near the Fram Strait[39]. One sample from the Fram Strait (PS122/4_49-14, 100 m) falls outside the expected f_{RIV}-ε_{Nd} data envelope (Fig. 4b, main text), likely due to localized input of Greenland meltwater (f_{RIV} = 4%) characterized by highly unradiogenic ε_{Nd} signatures[27], or due to non-conservative processes related to particulate inputs from Greenland (Supplementary Text 1). As this modification is locally confined, we excluded this sample from our water mass analysis and interpretation.

2.3 Sea ice meltwater or brine

Sea ice is an integral part of the surface Arctic Ocean. Its formation and melting lead to significant changes in the water column, affecting both salinity and tracer distributions. During sea ice formation, brine rejection increases salinity in the underlying water, while melting introduces fresh water, diluting surface waters and influencing stratification. We account for these volumetric changes and their effects on tracers by including sea ice meltwater (SIM; negative values are proportional to the subsequent addition of brines to the water column) in our water mass analysis. The ranges in SIM endmember values are derived from the observed variations in our pooled sea ice samples (see Methods section of the main text). These values are consistent with previous data (for salinity and $\delta^{18}O$, see Bauch et al.[30]; for ε_{Nd} and [Nd], see Laukert et al.[27,40]. This inclusion allows for a more precise understanding of the influence of sea ice on both the water mass composition and the tracers within the Arctic system.

2.4 Pacific-sourced water

Tracing Pacific water in the Arctic Ocean is critical due to its unique physicochemical properties but highly challenging due to the lack of suitable tracers. The small $\delta^{18}O$ difference between Atlantic (0.3 ‰) and Pacific (−1.1 ‰) waters prevents the use of stable oxygen isotopes to distinguish between the two. As a result, $\delta^{18}O$ is mainly applied to differentiate between marine and meteoric waters when other tracers are not available[30]. To overcome this limitation, researchers have traditionally relied on differences in macronutrient ratios between Atlantic and Pacific waters. Since these differences were first reported[35,41,42], numerous studies have applied mass balance calculations based on empirical nutrient ratios to trace Pacific water pathways through the Arctic Ocean[30,35,43-46]. In the Fram Strait—a key gateway between the Arctic and North Atlantic—Pacific water content has frequently been estimated using the nitrate-to-phosphate ratio (N/P)[5,39,47-55]. Some of these studies have observed significant interannual variability in these estimates, with Pacific water fractions ranging from nearly undiluted to nearly absent. While these

fluctuations reflect considerable changes in water composition in the Fram Strait and upstream regions, including the TPD, they are unlikely to reflect changes in Pacific water content itself.

Emerging evidence indicates that nutrient-derived Pacific estimates may not exclusively, or even primarily, indicate the presence of Pacific water. Instead, they could be heavily influenced by biogeochemical processes occurring outside the Chukchi Sea, such as bacterial denitrification in the shallow sediments of the East Siberian[56] and Laptev[57-59] seas. This process reduces nitrate relative to phosphate, leading to overestimations of Pacific water fractions[30,60,61]. Compounding this issue is an incomplete understanding of nitrate and phosphate cycling in the Arctic Ocean, which complicates efforts to account for non-conservative nutrient behaviors[30,60-63]. Consequently, discrepancies of up to 60% in the central Arctic Ocean[46] and 40% in the Fram Strait[5] have been reported between different nutrient-based Pacific water estimates. These inaccuracies lead to significant misrepresentations of the Arctic Ocean freshwater budget, resulting in misleading conclusions such as Pacific water export exceeding import, the transformation of Atlantic into Pacific water, and the presence of Pacific water at depths greater than 500 m[61]. Given the crucial role of the Fram Strait in Arctic freshwater fluxes and its position downstream of the TPD, these findings are vital to our study. They challenge the prevailing nutrient-based perspective on substantial Pacific water advection to the Fram Strait and, consequently, further upstream along the TPD. More recent, detailed analyses of nutrient relationships support an alternative scenario in which Pacific water is absent in this region. For instance, Willcox et al.[64] found no evidence of Pacific water advection to the Fram Strait, despite high nutrient-based Pacific fractions. Instead, they attributed the water column properties to inputs from the Laptev Sea and the Lena River. The overestimation of Pacific water is likely associated with phosphate enrichment beneath the Lena plume, driven by riverine inputs and winter processes[59]. The link between high Pacific fractions and Lena River inputs persists along the TPD[17] and in the Fram Strait[5], suggesting that changes in nutrient dynamics along this pathway are driven by variable Lena water advection and physicochemical processes in the Lena estuary rather than Pacific water advection.

A recent analysis of decadal mean geostrophic pathways[65] reinforces the conclusion that Pacific water is not present in the Eurasian Basin. Instead, this study shows that it is separated from Eurasian-sourced waters by a distinct front along the northern side of the Mendeleev and Alpha ridges and is exported from the Arctic Ocean exclusively through the Amundsen and M'Clure straits. Summer Pacific water, which spreads at depths relevant to our study (60-100 m[65]), comprises less than 30% of the water column as it enters the Amerasian Basin on the eastern side of the Chukchi Plateau[66]. Even considering strong sub-decadal (e.g., interannual) variability in geostrophic flow within the open Arctic Ocean, including potential frontal shifts, this summer Pacific water would likely be further diluted on its journey to the Eurasian Basin, resulting in Pacific water fractions well below 30%. Conductivity-temperature-depth profiles from the MOSAiC drift support these findings, as no distinct temperature minima or maxima were observed at salinities below 34, which would indicate significant advection of Pacific water. A quantitative water mass analysis that includes Pacific water alongside AW and the two riverine endmembers is not feasible due to the challenges of balancing Nd concentrations, which introduce significant sensitivity issues[17]. Similarly, trends observed in the ε_{Nd}-[Nd] space remain inconclusive regarding the contribution of Pacific water, as they can be interpreted as either an admixture of Pacific water or Yenisei/Ob water, both of which exhibit nearly identical ε_{Nd} signatures[17]. However, we can substitute the Yenisei/Ob endmember with Pacific water in our analysis to simulate an unlikely scenario where Pacific water, rather than Yenisei/Ob water, accounts for the more radiogenic band observed in our study and by Paffrath et al.[17]. This allows us to estimate the amount of Pacific water needed to account for this signal. Our analysis suggests Pacific water fractions of up to 70% at the surface and 50% at 100 m depth, which contradicts

the expected maximum contribution of 30% at depths between 60 and 100 m. Since this signal cannot be fully attributed to Pacific water, it likely reflects contributions from Yenisei/Ob waters, as the more radiogenic band presents a continuous signal extending to the surface, indicating a single water mass rather than two distinct ones with similar ε_{Nd} signatures. Nevertheless, even if partial admixture of Pacific water (<30%) at greater depths (60–100 m) were to occur, the high surface fractions would still reflect the advection of Yenisei/Ob water. Therefore, our conclusions regarding sea ice–ocean interactions in the surface layer remain valid regardless of the scenario.

The Nd concentrations and REE distribution patterns further support these observations, indicating the absence of Pacific water in our study area. The strong correlation between [Nd] and f_{RIV} emphasizes the predominant mixing of AW with river water, while the presence of substantial amounts of Pacific water with an [Nd] of 30 pmol kg^{-1} is not evident (Fig. 4a, main text). Extrapolating [Nd] at f_{RIV} = 100% yields 194 pmol kg^{-1}, closely aligning with estimates for the Siberian river endmember (210 pmol kg^{-1}; discharge-weighted [Nd] of Lena and Yenisei/Ob river water after a 75% Nd loss in the estuary), further indicating a two-component mixing between Siberian river water and AW as the sole marine endmember. The mixing of Yenisei/Ob and Lena river waters with AW is primarily driven by density differences, resulting in a gradual merging of these river waters with AW. This gradual integration and the very similar [Nd] endmember values of the two river systems result in a linear [Nd]-f_{RIV} relationship. The observed weaker correlation between [Nd] and f_{RIV} in the 2015 summer data has been attributed to a broader sampling area encompassing regions beyond the influence of the TPD, including the Makarov Basin, where admixture of Pacific water is more likely[17]. The absence of Pacific water in our study region is further supported by a comparison of REE distribution patterns between the Canada and Eurasian basins. In contrast to the Eurasian Basin, the water column in the Canada Basin, dominated by Pacific waters, exhibits stronger depletion of light REEs (LREEs) compared to heavy REEs (HREEs), leading to higher PAAS-normalized HREE/LREE ratios at lower salinities[25]. Such characteristics are absent in the surface waters encountered during the MOSAiC drift (Supplementary Fig. S6). Discrepancies in the lower HREE/LREE values (reaching ~2.5) reported by Paffrath et al.[17] remain unexplained for some deeper samples, but these are also not reproduced by our data (Supplementary Fig. S6). Moreover, considering the expected influence of Pacific water, higher rather than lower HREE/LREE ratios would be expected. Collectively, these observations strongly suggest that there was no significant influence of Pacific water in our study region during the MOSAiC expedition. This finding allows us to confidently exclude Pacific water from our water mass analysis.

Supplementary Text 3:

Effects of brine spillage during sampling, small-scale heterogeneity, and permeability changes

Comparison of the $\delta^{18}O$ values between the pooled samples of nine first-year ice (FYI) cores and the samples of one FYI core taken on the same day (April 8, 2020) and at the exact same location from the main coring site (MCS) reveals only subtle differences within the upper 20 cm, with pooled samples showing slightly lighter values by approximately 0.25 ‰ (Supplementary Fig. S7). However, notable discrepancies emerge in the distribution of the brine-associated parameters, i.e. bulk salinity and Nd concentration ([Nd]). Specifically, a shift in the 70−80 cm interval toward lower bulk salinity in the individual FYI core indicates that brine leaked out during sampling of this core. A more pronounced deviation from our pooled FYI profile is evident in the central part of a FYI core recovered from the MCS on April 6, 2020 (Supplementary Fig. S7). The higher bulk salinities in this core[67] cannot be attributed to brine leakage during sampling but may result from a very inhomogeneous brine distribution. This hypothesis is supported by the [Nd] distribution in the individual FYI core, which punctually exhibits elevated concentrations (40−50 cm) and shows less agreement with the seawater-derived $\delta^{18}O$ profile than the [Nd] profile from the pooled FYI samples (Supplementary Fig. S7). Notably, this discrepancy is not reflected in any of the $\delta^{18}O$ MOSAiC profiles[68], as most of the $\delta^{18}O$ signal originates from the ice crystal lattice and is therefore not influenced by brine-associated processes. However, our data demonstrate that the effects of both brine spillage and inhomogeneous brine distribution on the bulk salinity and [Nd] profiles, revealed by different sampling strategies, can be minimized by using pooled FYI samples. This also suggests that small-scale inhomogeneities do not significantly affect the distribution and dispersion of brine-associated substances on a larger scale.

Considering only the FYI profiles of the pooled ice cores, subtle differences in parameter distribution were observed despite common trends. The distribution pattern of bulk salinity, [Nd], ε_{Nd} and the HREE/LREE ratio exhibits an offset of approximately 10 cm from that of $\delta^{18}O$ (Fig. 5, main text). This offset could also be somewhat larger, which, however, cannot be narrowed down any further due to the sampling resolution of 10 cm. Adjusting the profiles of these parameters upwards by 10 cm results in much stronger correlations between them and $\delta^{18}O$ ($R^2 = 0.6$ for bulk salinity and ε_{Nd}, 0.8 for [Nd], with $p < 0.05$), compared to the initially weak correlations ($R^2 = 0.2$ for bulk salinity, 0.4 for [Nd], and 0.3 for ε_{Nd}, with $p < 0.05$). The high salinity of 5.5 at the bottom of the FYI was neglected, as it was probably caused by continuous exchange with underlying seawater due to the high permeability of the sea ice in the lowest centimeters (possibly <5 cm in the winter FYI[69]). Once again, this comparison demonstrates that salt and REEs accumulate in the liquid brine and are thus more susceptible to permeability changes, even in sea ice intervals further away from the ice–ocean interface, contrasting with oxygen isotope signatures of water that are mainly integrated into the ice crystal lattice[27,70]. Initially, higher permeability facilitates desalination through gravity drainage, resulting in a decrease in bulk salinity in the FYI to values between 3.4 and 4.7 (excluding the high value in the lowermost 10 cm) and REE concentrations well below those of the parental seawater, ranging from 1.8 to 3.4 pmol kg^{-1} for [Nd]. Conversely, as sea ice ages, decreasing permeability restricts the natural convection of remaining brines and reduces pore size, effectively isolating the pores from regular brine movement and preserving their original solute content until the melting season[69]. Restricted brine convection after initial desalination may thus have caused a limited but notable downward migration of salt and REEs, contributing to the observed offset between $\delta^{18}O$ and the brine-associated parameters, including ε_{Nd} due to its association with [Nd]. However, this process does not significantly affect the original distributions and thus is in line with the dominant influence of the parental seawater composition on these parameters.

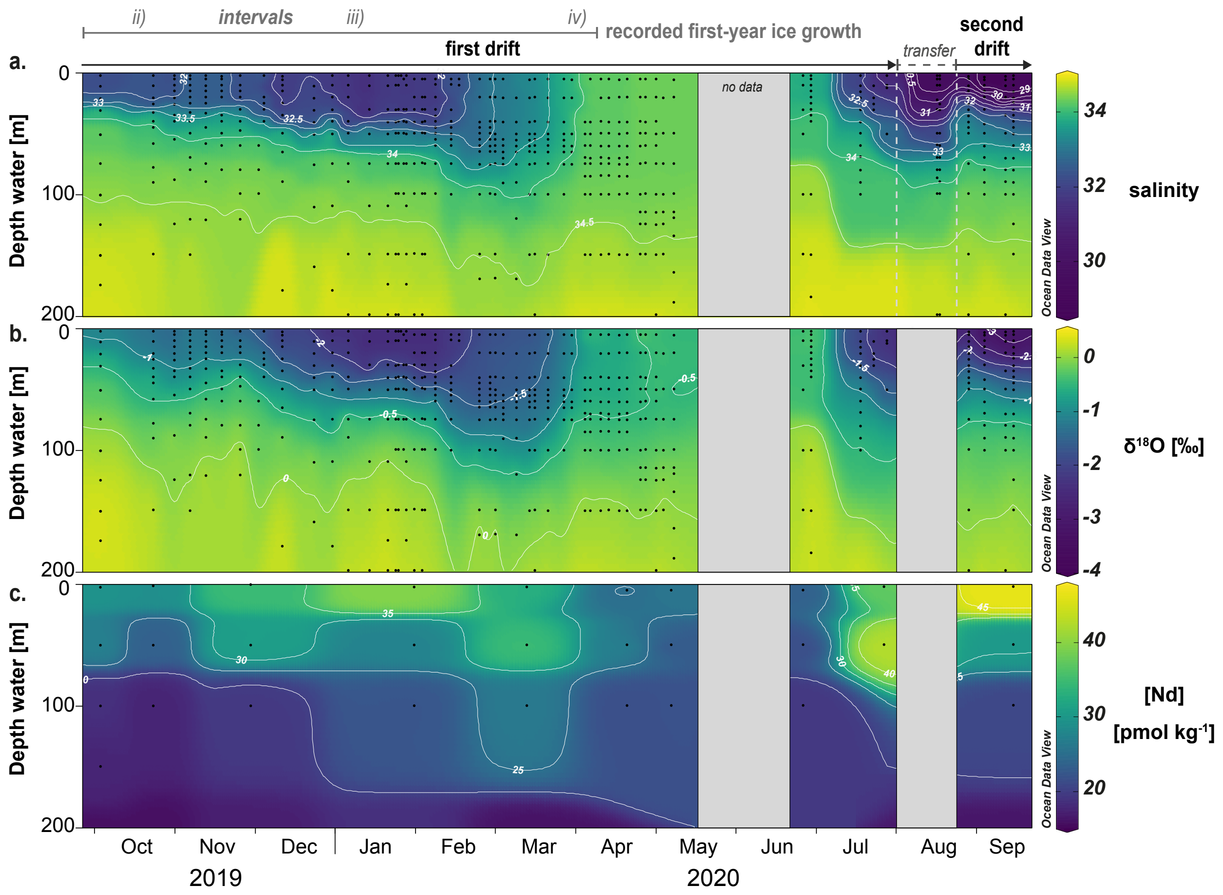

Supplementary Fig. S1: Sections of **a** salinity[71,72], **b** $\delta^{18}O$[73] and **c** neodymium concentrations ([Nd])[74]. The sections were created using ODV[75] and modified manually.

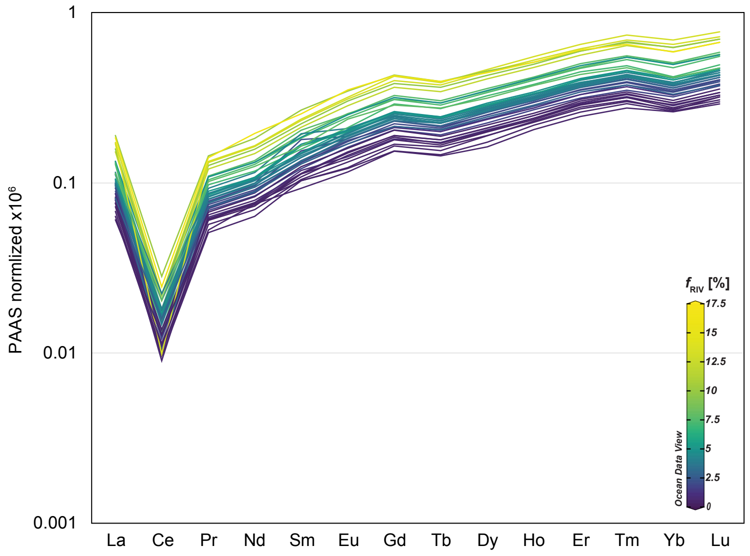

Supplementary Fig. S2: Seawater PAAS-normalized[76] REE distribution patterns[74] compared to the river water fraction (f_{RIV}, in percent) calculated based on salinity[71,72] and $\delta^{18}O$[73] (see Methods section of the main text).

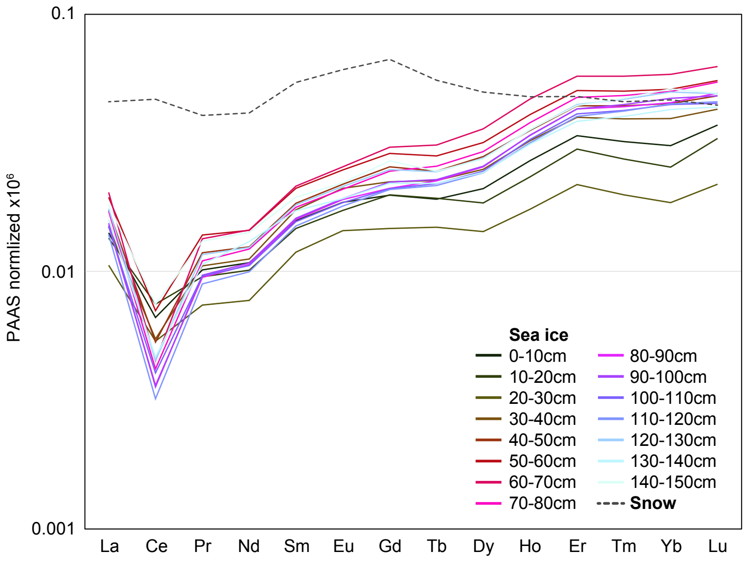

Supplementary Fig. S3: Sea ice and snow PAAS-normalized[76] REE distribution patterns[77].

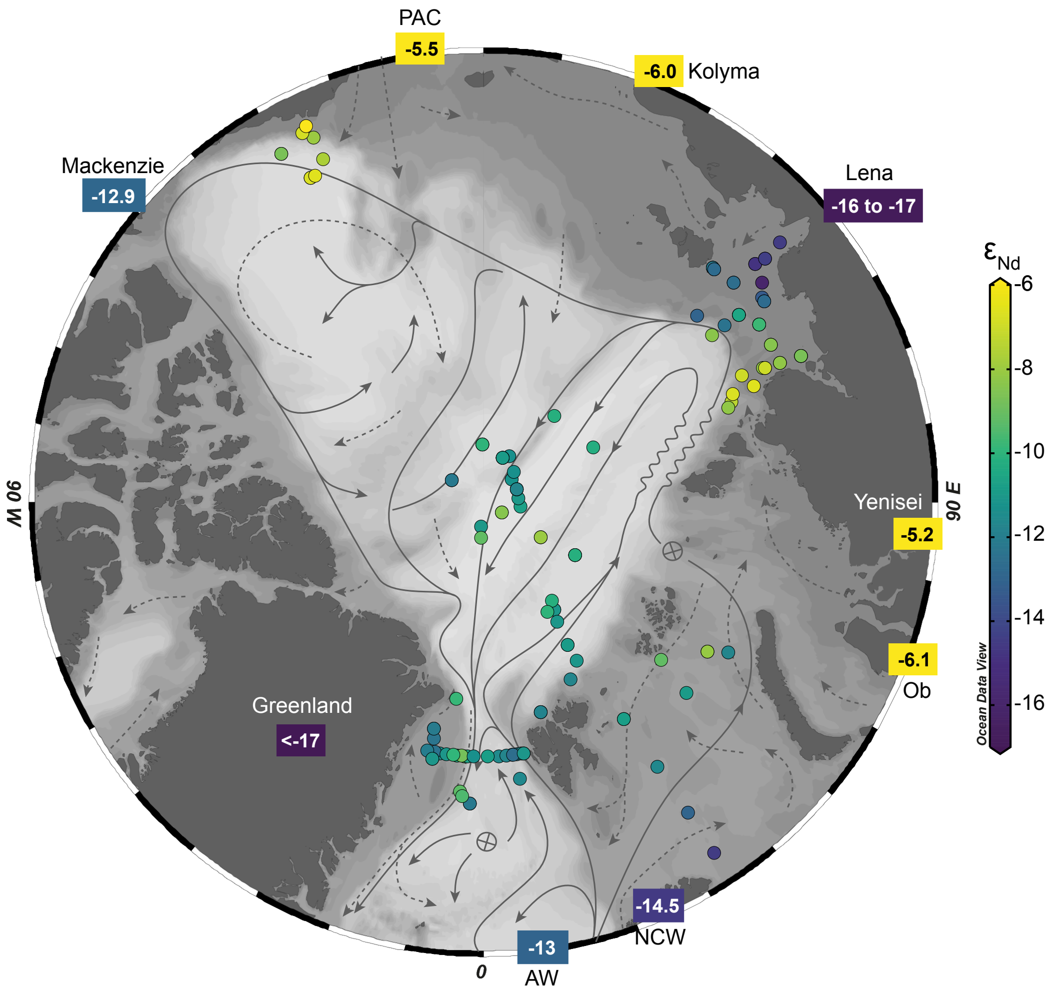

Supplementary Fig. S4: Bathymetric map of the Arctic Ocean with major Nd sources (AW: Atlantic Water, PAC: Pacific Water, NCW: Norwegian Coastal Water, and the major Arctic rivers Ob, Yenisei, Lena, Kolyma, and Mackenzie) and their ε_{Nd} signatures, as well as published surface seawater ε_{Nd} data[4-6,8,15-18,78]. Additionally, the general circulation pattern of surface waters (dashed grey arrows) and AW (grey arrows) is shown (modified after Rudels et al.[79]).

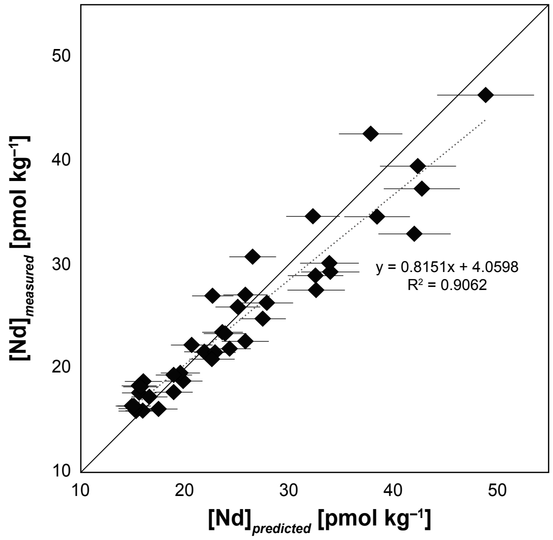

Fig. S5: Comparison of measured Nd concentrations ([Nd]*measured*) of the samples[74] with calculated values based on our water mass analysis ([Nd]*predicted*) (see Methods section of the main text). The slight deviation to lower [Nd]*measured* values at higher concentrations is attributed to increased rates of estuarine Nd removal or to minor contributions from Siberian rivers with lower [Nd].

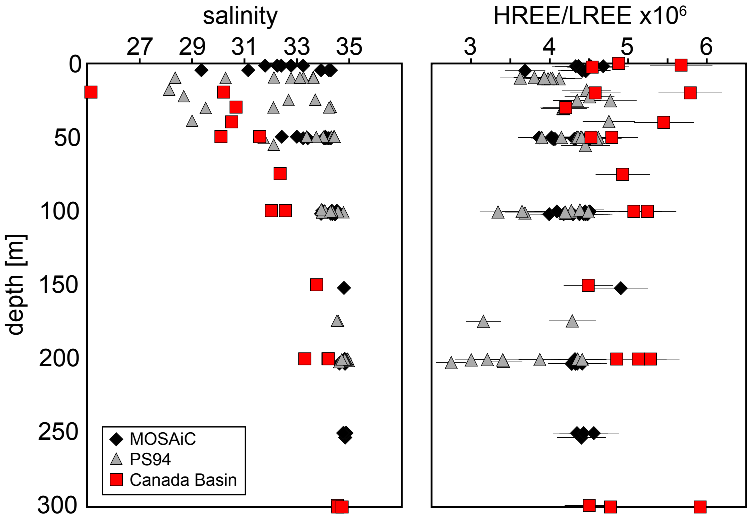

Supplementary Fig. S6: Comparison of salinity and PAAS-normalized[76] HREE/LREE distribution as a function of water depth between the Canada Basin of the Arctic Ocean[25] and the open Eurasian Arctic Ocean (MOSAiC: this study[74]; PS94:[17]).

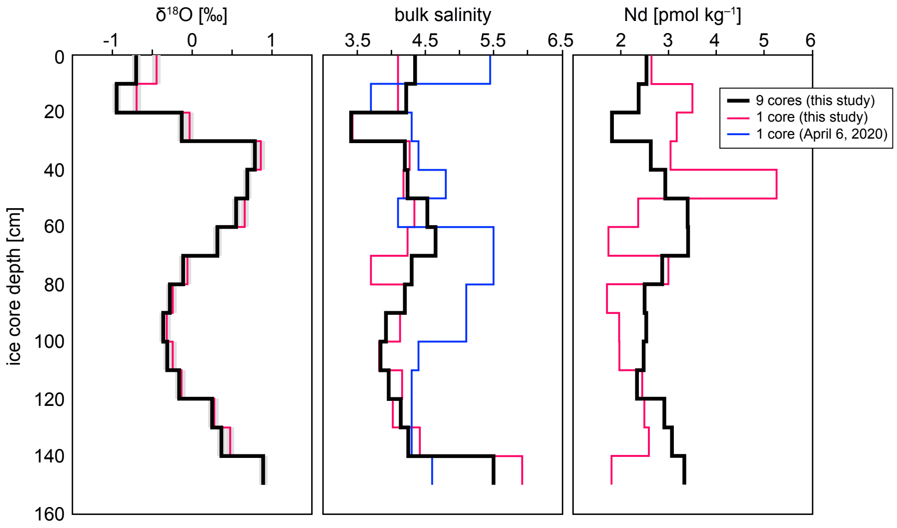

Supplementary Fig. S7: Comparison of $\delta^{18}O$ values, salinity and Nd concentration between pooled samples from nine FYI cores (this study[77]), samples from one FYI core (this study[77]), and samples from one FYI core collected on April 6, 2020, from the MCS[67] (salinity only).

Table S1: Endmember compositions used for the water mass analysis. Ranges are provided where available; otherwise, analytical uncertainties are reported in brackets.

	**salinity**	**$\delta^{18}O$ (‰)**	**ε_{Nd}**	**[Nd] (pmol kg^{-1})**
**Atlantic water**	35.02 – 35.09	+0.27 to +0.33	−12.4 to −11.7	11 – 15.9
**Pacific water**	32.7 (0.005)	−1.1 (0.05)	−5.5 (0.4)	30 (0.5)
**Lena**	0 (0.005)	−21.5 to −19.5	−16.7 to −15	120 – 223
**Yenisei/Ob**	0 (0.005)	−18.4 to −14.9	−6.1 to −5.2	193 – 289
**Sea ice meltwater**	3.41 – 5.5	−0.95 to +0.89	−9.34 to −10.76	1.81 – 3.41

References

1. Jeandel, C. Overview of the mechanisms that could explain the 'Boundary Exchange' at the land–ocean contact. *Phil. Trans. R. Soc. A* **374**, 20150287 (2016). https://doi.org/10.1098/rsta.2015.0287
2. Rousseau, T. C. C. *et al.* Rapid neodymium release to marine waters from lithogenic sediments in the Amazon estuary. *Nat. Commun.* **6**, 7592 (2015). https://doi.org/10.1038/ncomms8592
3. Xu, A. *et al.* Overlooked riverine contributions of dissolved neodymium and hafnium to the Amazon estuary and oceans. *Nat. Commun.* **14**, 4156 (2023). https://doi.org/10.1038/s41467-023-39922-3
4. Porcelli, D. *et al.* The distribution of neodymium isotopes in Arctic Ocean basins. *Geochim. Cosmochim. Acta* **73**, 2645–2659 (2009). https://doi.org/10.1016/j.gca.2008.11.046
5. Laukert, G. *et al.* Ocean circulation and freshwater pathways in the Arctic Mediterranean based on a combined Nd isotope, REE and oxygen isotope section across Fram Strait. *Geochim. Cosmochim. Acta* **202**, 285–309 (2017). https://doi.org/10.1016/j.gca.2016.12.028
6. Laukert, G. *et al.* Transport and transformation of riverine neodymium isotope and rare earth element signatures in high latitude estuaries: A case study from the Laptev Sea. *Earth Planet. Sci. Lett.* **477**, 205–217 (2017). https://doi.org/10.1016/j.epsl.2017.08.010
7. Pokrovsky, O. S. *et al.* Fate of colloids during estuarine mixing in the Arctic. *Ocean Sci.* **10**, 107–125 (2014). https://doi.org/10.5194/os-10-107-2014
8. Laukert, G. *et al.* Water mass transformation in the Barents Sea inferred from radiogenic neodymium isotopes, rare earth elements and stable oxygen isotopes. *Chem. Geol.* **511**, 416–430 (2019). https://doi.org/10.1016/j.chemgeo.2018.10.002
9. Gordeev, V. V. Fluvial sediment flux to the Arctic Ocean. *Geomorphology* **80**, 94–104 (2006). https://doi.org/10.1016/j.geomorph.2005.09.008
10. Gordeev, V. V. *et al.* A reassessment of the Eurasian river input of water, sediment, major elements, and nutrients to the Arctic Ocean. *Am. J. Sci.* **296**, 664–691 (1996). https://doi.org/10.2475/ajs.296.6.664
11. Dittmar, T. & Kattner, G. The biogeochemistry of the river and shelf ecosystem of the Arctic Ocean: A review. *Mar. Chem.* **83**, 103–120 (2003). https://doi.org/10.1016/s0304-4203(03)00105-1
12. Tepe, N. & Bau, M. Behavior of rare earth elements and yttrium during simulation of arctic estuarine mixing between glacial-fed river waters and seawater. *Chem. Geol.* **438**, 134–145 (2016). https://doi.org/10.1016/j.chemgeo.2016.06.001
13. Merschel, G., Bau, M., Dantas, E. L. Contrasting impact of organic and inorganic nanoparticles and colloids on particle-reactive elements in tropical estuaries: An experimental study. *Geochim. Cosmochim. Acta* **197**, 1–13 (2017). https://doi.org/10.1016/j.gca.2016.09.041
14. Schmitt, W. Application of the Sm–Nd Isotope System to the Late Quaternary Paleoceanography of the Yermak Plateau. Dissertation, Ludwig-Maximilians-Universität München (2007).
15. Zimmermann, B. *et al.* Hafnium isotopes in Arctic Ocean water. *Geochim. Cosmochim. Acta* **73**, 3218–3233 (2009). https://doi.org/10.1016/j.gca.2009.02.028
16. Persson, P. O. *et al.* The influence of Lena River water inflow and shelf sediment-sea water exchange for the Nd isotopic composition in the Laptev Sea and Arctic Ocean. *Geophys. Res. Abstr.* 13 (2011).
17. Paffrath, R. *et al.* Separating individual contributions of major Siberian rivers in the Transpolar Drift of the Arctic Ocean. *Sci. Rep.* **11**, 8216 (2021). https://doi.org/10.1038/s41598-021-86948-y
18. Charette, M. A. *et al.* Coastal ocean and shelf-sea biogeochemical cycling of trace elements and isotopes: lessons learned from GEOTRACES. *Phil. Trans. A Math. Phys. Eng. Sci.* **374**, 20160076 (2016). https://doi.org/10.1098/rsta.2016.0076
19. Pfirman, S. L. *et al.* Potential for rapid transport of contaminants from the Kara Sea. *Sci. Total Environ.* **202**, 111–122 (1997). https://doi.org/10.1016/S0048-9697(97)00108-3
20. Steele, M. *et al.* Circulation of summer Pacific halocline water in the Arctic Ocean. *J. Geophys. Res. Oceans* **109**, C2 (2004). https://doi.org/10.1029/2003JC002009
21. Schulz, K. *et al.* The Eurasian Arctic Ocean along the MOSAiC drift in 2019–2020: an interdisciplinary perspective on physical properties and processes. *Elem. Sci. Anth.* **12**, 1 (2024). https://doi.org/10.1525/elementa.2023.00114

22. Paffrath, R. *et al.* Seawater-particle interactions of rare earth elements and neodymium isotopes in the deep central Arctic Ocean. *J. Geophys. Res. Oceans* **126**, e2021JC017423 (2021). https://doi.org/10.1029/2021JC017423
23. van de Flierdt, T. *et al.* Neodymium in the oceans: A global database, a regional comparison and implications for palaeoceanographic research. *Phil. Trans. A Math. Phys. Eng. Sci.* **374** (2016). https://doi.org/10.1098/rsta.2015.0293
24. Charette, M. A. *et al.* Coastal ocean and shelf-sea biogeochemical cycling of trace elements and isotopes: lessons learned from GEOTRACES. *Mar. Chem.* **177**, 1–8 (2015). https://doi.org/10.1016/j.marchem.2015.06.011
25. Yang, J. & Haley, B. The profile of the rare earth elements in the Canada Basin, Arctic Ocean. *Geochem. Geophys. Geosyst.* **17**, 3241–3253 (2016). https://doi.org/10.1002/2016GC006412
26. Laukert, G. *et al.* Greenland-sourced freshwater traced by radiogenic neodymium isotopes and rare earth elements on the North-East Greenland Shelf. *Goldschmidt Abstracts*, 1419 (2018).
27. Laukert, G. *et al.* Neodymium isotopes trace marine provenance of Arctic sea ice. *Geochem. Perspect. Lett.* **22**, 10–15 (2022). https://doi.org/10.7185/geochemlet.2220
28. Rudels, B. *et al.* The interaction between waters from the Arctic Ocean and the Nordic Seas north of Fram Strait and along the East Greenland Current. *J. Mar. Syst.* **55**, 1–30 (2005). https://doi.org/10.1016/j.jmarsys.2004.06.008
29. Rosén, P.-O. *et al.* Ice export from the Laptev and East Siberian Sea derived from δ18O values. *J. Geophys. Res. Oceans* **120**, 5997–6007 (2015). https://doi.org/10.1002/2015JC010866
30. Bauch, D. *et al.* Origin of freshwater and polynya water in the Arctic Ocean halocline in summer 2007. *Prog. Oceanogr.* **91**, 482–495 (2011). https://doi.org/10.1016/j.pocean.2011.07.017
31. Östlund, H. G. & Hut, G. Arctic Ocean water mass balance from isotope data. *J. Geophys. Res.* **89** (1984). https://doi.org/10.1029/JC089iC04p06373
32. Rudels, B. *et al.* Circulation and transformation of Atlantic water in the Eurasian Basin and the contribution of the Fram Strait inflow branch to the Arctic Ocean heat budget. *Prog. Oceanogr.* **132**, 128–152 (2015). https://doi.org/10.1016/j.pocean.2014.04.003
33. Winkelbauer, S., Mayer, M., Seitner, V., Zsoter, E., Zuo, H. & Haimberger, L. Diagnostic evaluation of river discharge into the Arctic Ocean and its impact on oceanic volume transports. *Hydrol. Earth Syst. Sci.* **26**, 279–304 (2022). https://doi.org/10.5194/hess-26-279-2022
34. Cooper, L. W. *et al.* Flow-weighted values of runoff tracers from the six largest Arctic rivers. *Geophys. Res. Lett.* **35** (2008). https://doi.org/10.1029/2008GL035007
35. Ekwurzel, B. *et al.* River runoff, sea ice meltwater, and Pacific water distribution and mean residence times in the Arctic Ocean. *J. Geophys. Res. Oceans* **106**, 9075–9092 (2001). https://doi.org/10.1029/1999JC000024
36. Mulligan, R. P. & Perrie, W. Circulation and structure of the Mackenzie River plume in the coastal Arctic Ocean. *Cont. Shelf Res.* **177**, 59–68 (2019). https://doi.org/10.1016/j.csr.2019.04.001
37. Macdonald, R. W. *et al.* Connections among ice, runoff, and atmospheric forcing in the Beaufort Gyre. *Geophys. Res. Lett.* **26**, 2223–2226 (1999). https://doi.org/10.1029/1999GL900508
38. Van Straaten, C., Lique, C., Kolodziejcyk, N. The life cycle of low salinity lenses in the Arctic Ocean. *ESS Open Archive* (2024). https://doi.org/10.22541/essoar.172408092.25227238/v1
39. Stedmon, C. A. *et al.* Freshwater contribution estimation in East Greenland shelf waters. *J. Geophys. Res. Oceans* **120**, 1107–1117 (2015). https://doi.org/10.1002/2014JC010501
40. Laukert, G. *et al.* Pathways of Siberian freshwater in the Arctic Ocean. *Polarforschung* **87**, 3–13 (2017). https://doi.org/10.2312/polarforschung.87.1.3
41. Bauch, D. *et al.* Freshwater balance and sources in the Arctic Ocean inferred from H218O. *Prog. Oceanogr.* **35**, 53–80 (1995). https://doi.org/10.1016/0079-6611(95)00005-2
42. Jones, E. P. *et al.* Atlantic and Pacific waters in the upper Arctic Ocean. *Geophys. Res. Lett.* **25**, 765–768 (1998). https://doi.org/10.1029/98GL00464
43. Yamamoto-Kawai, M. *et al.* Freshwater budget of the Canada Basin, Arctic Ocean. *J. Geophys. Res.* **113** (2008). https://doi.org/10.1029/2006JC003858

44. Jones, E. P. *et al.* Freshwater sources across Arctic Ocean basins. *J. Geophys. Res. Oceans* **113** (2008). https://doi.org/10.1029/2007JC004124
45. Newton, R. *et al.* Canadian Basin freshwater sources and changes. *J. Geophys. Res. Oceans* **118**, 2133–2154 (2013). https://doi.org/10.1002/jgrc.20101
46. Alkire, M. B. *et al.* Variability of meteoric water, sea-ice melt, and Pacific water in the Arctic Ocean. *J. Geophys. Res. Oceans* **120**, 1573–1598 (2015). https://doi.org/10.1002/2014JC010023
47. de Steur, L. *et al.* Freshwater composition east of Greenland. *Geophys. Res. Lett.* **42**, 2326–2332 (2015). https://doi.org/10.1002/2014GL062759
48. Dodd, P. A. *et al.* Freshwater export in the East Greenland Current. *Geophys. Res. Lett.* **36** (2009). https://doi.org/10.1029/2009GL039663
49. Dodd, P. A. *et al.* Freshwater composition of Fram Strait outflow. *J. Geophys. Res. Oceans* **117** (2012). https://doi.org/10.1029/2012JC008011
50. Falck, E. Atlantic and Pacific waters in the Northeast Water Polynya. *Polar Res.* **20**, 193–200 (2001). https://doi.org/10.1111/j.1751-8369.2001.tb00056.x
51. Falck, E. *et al.* Disappearance of Pacific Water in the northwestern Fram Strait. *Geophys. Res. Lett* **32** (2005). https://doi.org/10.1029/2005GL023400
52. Jones, E. P. *et al.* Sources and distribution of fresh water in the East Greenland Current. *Prog. Oceanogr.* **78**, 37–52 (2008). https://doi.org/10.1016/j.pocean.2007.06.003
53. Rabe, B. *et al.* Liquid export of Arctic freshwater through the Fram Strait. *Ocean Sci.* **9**, 91–109 (2013). https://doi.org/10.5194/os-9-91-2013
54. Sutherland, D. A. *et al.* Freshwater composition off southeast Greenland and its link to the Arctic Ocean. *J. Geophys. Res. Oceans* **114** (2009). https://doi.org/10.1029/2008JC004808
55. Taylor, J. R. *et al.* Dissolved barium as a tracer in the Arctic Ocean. *J. Geophys. Res. Oceans* **108** (2003). https://doi.org/10.1029/2002JC001635
56. Anderson, L. G. *et al.* Source and formation of the Arctic Ocean halocline. *J. Geophys. Res. Oceans* **118**, 410–421 (2013). https://doi.org/10.1029/2012JC008291
57. Nitishinsky, M. *et al.* Inorganic carbon and nutrient fluxes on the Arctic Shelf. *Cont. Shelf Res.* **27**, 1584–1599 (2007). https://doi.org/10.1016/j.csr.2007.01.019
58. Sun, X. *et al.* Benthic nutrient fluxes in the Laptev and East Siberian shelf seas. *Glob. Biogeochem. Cycles* **35** (2021). https://doi.org/10.1029/2020GB006849
59. Laukert, G. *et al.* Nutrient and silicon isotope dynamics in the Laptev Sea. *Glob. Biogeochem. Cycles* **36** (2022). https://doi.org/10.1029/2022GB007316
60. Alkire, M. B. *et al.* Atlantic/Pacific front identification in the Arctic Ocean. *Geophys. Res. Lett.* **46**, 3843–3852 (2019). https://doi.org/10.1029/2018GL081837
61. Forryan, A. *et al.* Arctic freshwater fluxes and tracer budgets. *Cryosphere* **13**, 2111–2131 (2019). https://doi.org/10.5194/tc-13-2111-2019
62. Chang, B. X. & Devol, A. H. Seasonal patterns of denitrification rates in the Chukchi Sea. *Deep-Sea Res. Pt. II* **56**, 1339–1350 (2009). https://doi.org/10.1016/j.dsr2.2008.10.024
63. Torres-Valdés, S. *et al.* Dissolved organic nutrients in the Arctic Ocean. *Geophys. Res. Lett.* **43**, 6418–6426 (2016). https://doi.org/10.1002/2016GL069245
64. Willcox, E. W. *et al.* Water masses on the Northeast Greenland shelf. *J. Geophys. Res. Oceans* **128**, e2022JC019052 (2023). https://doi.org/10.1029/2022JC019052
65. Planat, N. *et al.* Geostrophic pathways of Pacific and Atlantic waters in the Arctic Amerasian Basin. *ESS Open Archive* (2024). https://doi.org/10.22541/essoar.172124890.02099361/v1
66. Lin, P. *et al.* Fate of warm Pacific water in the Arctic basin. *Geophys. Res. Lett.* **48**, e2021GL094693 (2021). https://doi.org/10.1029/2021GL094693
67. Angelopoulos, M. *et al.* Properties of different Arctic ice types during MOSAiC. *Front. Earth Sci.* **10** (2022). https://doi.org/10.3389/feart.2022.864523
68. Mellat, M. *et al.* Isotopic signatures during MOSAiC. *Elem. Sci. Anth.* **12** (2024). https://doi.org/10.1525/elementa.2023.00078
69. Petrich, C. & Eicken, H. Overview of sea ice growth and properties. *Sea Ice*, 1–41 (2017).

70. Meiners, K. M. & Michel, C. Dynamics of nutrients and dissolved organic matter in sea ice. *Sea Ice*, 415–432 (2017). https://doi.org/10.1002/9781118778371.ch17
71. Tippenhauer, S. *et al.* Physical oceanography water bottle samples based on ship CTD during POLARSTERN cruise PS122. *PANGAEA* (2023). https://doi.org/10.1594/PANGAEA.959965
72. Tippenhauer, S. *et al.* Physical oceanography water bottle samples based on Ocean City CTD during POLARSTERN cruise PS122. *PANGAEA* (2023). https://doi.org/10.1525/elementa.2021.00062
73. Bauch, D., Andersen, N., Damm, E., D'Angelo, A. & Daiki, N. Stable water isotopes of seawater samples from the MOSAiC expedition 2019/2020. *PANGAEA* (2024). https://doi.org/10.1594/PANGAEA.966184
74. Laukert, G. *et al.* Dissolved radiogenic neodymium isotopes, stable oxygen isotopes and rare earth element concentrations of water bottle samples collected during MOSAiC. *PANGAEA* (2024). https://doi.org/10.1594/PANGAEA.966223
75. Schlitzer, R. Ocean Data View. http://odv.awi.de. (2024).
76. McLennan S. M. (2001) Relationships between the trace element composition of sedimentary rocks and upper continental crust. *Geochem. Geophys. Geosyst.* **2**. https://doi.org/10.1029/2000GC000109
77. Laukert, G., Damm, E., Simões Pereira, P., Bauch, D. & Hathorne, E. C. Dissolved radiogenic neodymium, stable oxygen isotopes and rare earth element concentrations of sea ice and snow samples collected during MOSAiC leg 3. *PANGAEA* (2024). https://doi.org/10.1594/PANGAEA.966225
78. Andersson, P. S. *et al.* Neodymium isotopes in seawater from Arctic gateways. *Geochim. Cosmochim. Acta* **72**, 2854–2867 (2008). https://doi.org/10.1016/j.gca.2008.04.008
79. Rudels, B. *et al.* Observations in the Ocean. *Arctic Climate Change* (Springer), 117–198 (2012).
